# Left Ventricular Hypertrophy After Renal Transplantation: Systematic Review and Meta-analysis

**DOI:** 10.1097/TXD.0000000000001647

**Published:** 2024-05-17

**Authors:** Zhejia Tian, Kai Bergmann, Jessica Kaufeld, Kai Schmidt-Ott, Anette Melk, Bernhard M.W. Schmidt

**Affiliations:** 1 Department of Nephrology and Hypertension, Hannover Medical School, Hannover, Germany.; 2 Department of Pediatric Kidney, Liver and Metabolic Diseases, Hannover Medical School, Hannover, Germany.

## Abstract

**Background.:**

Left ventricular hypertrophy (LVH) in patients with end stage renal disease undergoing renal replacement is linked to an increased risk for cardiovascular diseases. Dialysis does not completely prevent or correct this abnormality, and the evidence for kidney transplantation (KT) varies. This analysis aims to explore the relationship between KT and LVH.

**Methods.:**

MEDLINE and Scopus were systematically searched in October 2023. All cross-sectional and longitudinal studies that fulfilled our inclusion criteria were included. Outcome was left ventricular mass index (LVMI) changes. We conducted a meta-analysis using a random effects model. Meta-regression was applied to examine the LVMI changes dependent on various covariates. Sensitivity analysis was used to handle outlying or influential studies and address publication bias.

**Results.:**

From 7416 records, 46 studies met the inclusion criteria with 4122 included participants in total. Longitudinal studies demonstrated an improvement of LVMI after KT −0.44 g/m^2^ (−0.60 to −0.28). Blood pressure was identified as a predictor of LVMI change. A younger age at the time of KT and well-controlled anemia were also associated with regression of LVH. In studies longitudinally comparing patients on dialysis and renal transplant recipients, no difference was detected −0.09 g/m^2^ (−0.33 to 0.16). Meta-regression using changes of systolic blood pressure as a covariate showed an association between higher blood pressure and an increase in LVMI, regardless of the modality of renal replacement treatment.

**Conclusions.:**

In conclusion, our results indicated a potential cardiovascular benefit, defined as the regression of LVH, after KT. This benefit was primarily attributed to improved blood pressure control rather than the transplantation itself.

## Graphical abstract

**Figure:**
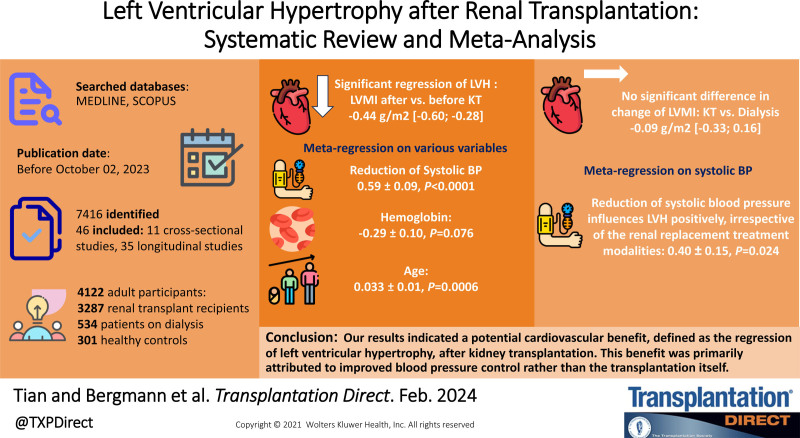

Renal transplantation is the treatment of choice of end-stage kidney disease. Besides an improvement of quality of live, patient survival is improved after renal transplantation. Nevertheless, mortality is increased compared with the general population. The most prominent cause for this increased mortality is the elevated cardiovascular risk in the transplant population.^1^

Left ventricular hypertrophy (LVH) is a strong predictor of cardiovascular events.^2-5^ Notably, it has been consistently demonstrated that a reduction in left ventricular mass (LVM) is correlated to a decrease in cardiovascular risk.^6,7^ This correlation stands out as the most robust among all cardiac and vascular parameters used for risk estimation.^8^ Therefore, the assessment of LVM is the corner stone of cardiovascular risk assessment and is used for longitudinal assessment of treatment success in clinical practice.

The aim of our analysis is to evaluate the evidence for a decrease of LVM after renal transplantation. A recent meta-analysis on LVM after renal transplantation^9^ focused on studies longitudinally comparing transplanted patients and patients on dialysis only, which provides only a very scarce data basis. Our analysis used a broader approach. We intended to include all available evidence into our analysis.

## MATERIALS AND METHODS

The study was registered in the PROSPERO database (CRD42023468541) and reported according to the PRISMA 2020 Statement (**Table S1, SDC**, http://links.lww.com/TXD/A657).

### Inclusion Criteria

#### Search Strategy

We searched MEDLINE and SCOPUS on October 02, 2023, using the following search terms: ((left ventricular mass) OR (left ventricular hypertrophy)) AND ((renal) OR (kidney)) AND transplant with publication year from January 1, 1901, to October 1, 2023.

#### Study Selection

The first two authors independently reviewed the titles and abstracts of retrieved articles for potential eligibility. Subsequently, they selected full-text articles by applying the predefined inclusion criteria. Disagreements were resolved by consulting a third author. We used an online research tool (Rayyan.ai) for initial screening of titles and abstracts. For selection of full-text articles, we used a reference management software (Mendeley, Elsevier).

We identified three types of studies: cross-sectional studies comparing renal transplant recipients with various control groups, longitudinal studies comparing renal transplant recipients before and after transplantation and longitudinal studies comparing LVM index (LVMI) changes in patients getting transplanted with patients remaining on dialysis.

### Data Collection and Quality Assessment

Two reviewers (Z.T. and K.B.) independently extracted data from included trials, resolving discrepancies through involvement of a third author (B.M.W.S.). The quality of the included trials was evaluated using a tool for assessing the risk of bias in cohort studies, developed by the CLARITY Group at McMaster University (https://www.distillersr.com/resources/methodological-resources/tool-to-assess-risk-of-bias-in-cohort-studies-distillersr). We applied the grading of recommendations, assessments, development and evaluation approach to evaluate the certainty of the evidence.^10^

### Outcomes

In longitudinal studies without a control group, the primary outcome was the changes in LVMI from baseline to follow-up in renal transplant recipients. The primary outcome in longitudinal studies involving renal transplant recipients and patients remaining on dialysis was recognized as the between-group differences in changes in LVMI from baseline to follow-up. The primary outcome was the differences in LVMI between patients after kidney transplantation (KT) and those remaining on dialysis or healthy participants.

### Data Analysis

We calculated mean difference (MD) of changes in LVMI between transplantation and control groups. Within-group Standardized MD (SMD) and SE of SMD were calculated for LVMI changes before and after KT in longitudinal studies. For data presented as median with first and third quartiles, we estimated the MD using the method of Luo et al^11^ and the SD according to Wan et al.^12^

R version 4.2.0 with *meta* package was used for all statistical analysis. We used the random effects model, as outlined by DerSimonian and Laird^13^ (4), to combine the results of the studies. Cochrane’s *Q*, Higgins and Thompson’s *I*^2,14^ and Tau2 were used for assessing heterogeneity between studies. The *I*^2^ value above 75% was considered as evidence of substantial heterogeneity. We used *dmetar* package to search for outlying studies, those with extreme effect size and influential studies, those that heavily influence the effect in one direction. We also applied a meta-regression model to elucidate the heterogeneity in terms of study-level covariates. We assessed publication bias by examining funnel plots symmetry and by conducting the Egger regression test. The Duval and Tweedie trim and fill method was used to adjust for funnel plot asymmetry.^15^

## RESULTS

**FIGURE 1.**
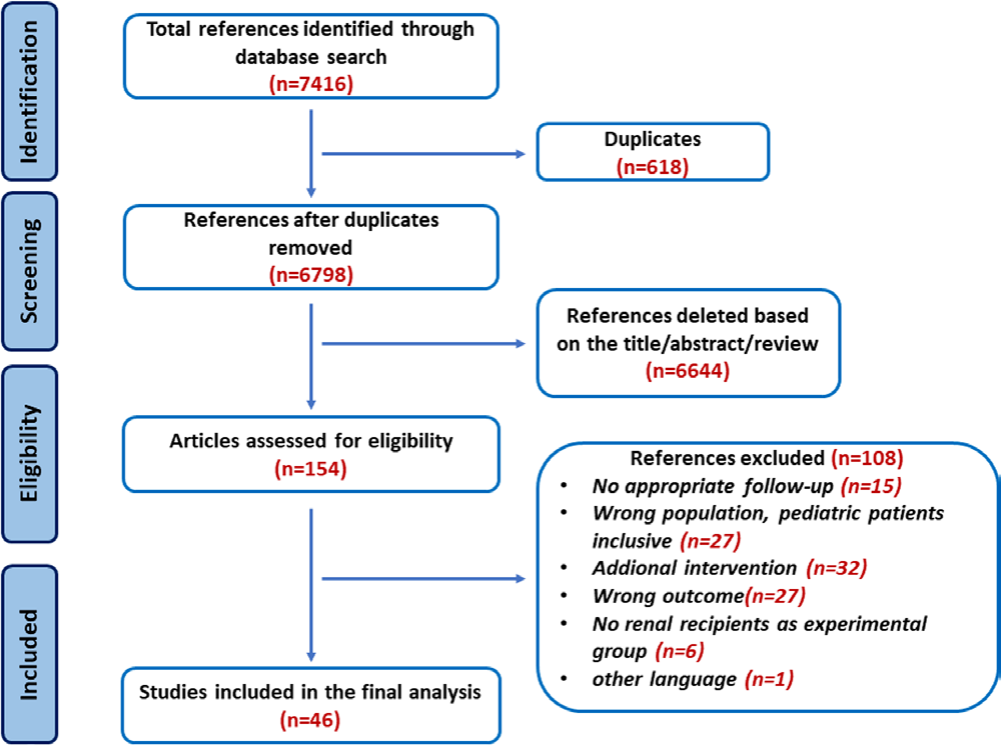
Flow diagram of study selection process.

Figure 1 displays the flow diagram illustrating the process of study selection. Initially, 7416 references were identified. Forty-six studies,^16–61^ involving 4122 participants with a mean age of 46.2 ± 7.6 y and a mean body mass index of 26.4 ± 10.3 kg/m^2^, met the inclusion criteria. Eleven studies were cross-sectional, and 35 were longitudinal. Among these participants, 3287 underwent KT, 534 remained on dialysis, and 301 served as healthy controls. Of all participants, 60.3% were male, and they had a mean systolic blood pressure of 139.5 ± 10.9 mm Hg. The baseline characteristics of included studies are summarized in Table 1.

**TABLE 1.** Baseline characteristics of included studies

Author	Year	Study type	Method	Control 1	Control 2	Duration of dialysis (mo)			1. M (wk)	2. M duration to KT (wk)	Participants (n)			Age (y)			Male (%)			SBP baseline (mm Hg)			SBP endpoint (mm Hg)			DBP baseline (mm Hg)			DBP endpoint (mm Hg)			GRADE certainty of the evidence
						KT	Control				KT	Control		KT	Control		KT	Control		KT	Control		KT	Control		KT	Control		KT	Control		
Ikaheimo	1982	2	2D Echo	—	—	—	—	—	18 (post-D) pre-KT	36	13	—	—	31	—	—	69	—	—	156	—	—	150	—	—	86	—	—	94	—	—	Very low
Larsson	1986	2	2D Echo		Healthy	22	—	—	Pre-KT	52	17		27	33	—	26	61		100	174	—	—	144	—	—	90	—	—	84	—	—	Very low
Hüting	1992	2	2D Echo	—	—		—	—	4 (pre-D) pre-KT	164	24	—	—	47	—	—	75	—	—	156	—	—	144	—	—	88	—	—	88	—	—	Very low
De Lima	1995	3	2D Echo	Dialysis (HD)	—	47	50	—	≥60 to 2.M pre-KT	73,2	16	36	—	41	44	—	47	50	—	155	155	—	153	137	—	97	94	—	97	84	—	Low
Parfrey	1995	2	2D Echo	—	—	—	—	—	≤48 pre-KT	188	102	—	—	51	—	—	72			144	—	—	134	—	—	84	—	—	84	—	—	Very low
Hernández	1997	2	2D Echo	—	—	29	—	—	0 (post-D) at KT	48	38	—	—	46	—	—	68	—	—	144	—	—	147	—	—	87	—	—	91	—	—	Low
Covic	1998	1	2D Echo	Dialysis (HD)	—	—	—	—	—	≥48	28	35	—	49	50	—	57	63	—	—	—	—	—	—	—	—	—	—	—	—	—	Very low
De Lima	1999	1	2D Echo	Dialysis (HD)	—	23	23	—	—	5	31	73	—	41	43	—	35	55	—	—	—	—	—	—	—	—	—	—	—	—	—	Very low
Suwelack	1999	1	2D Echo	—	Healthy	22	—	23	—	14.5	35	—	29	45	—	44	26	—	24	—	—	—	—	—	—	—	—	—	—	—	—	Very low
De Lima	2002	2	2D Echo	—	—	23	—	—	2.5 (post-KT)	48	22	—	—	41	—	—	46	—	—	138	—	—	140	—	—	85	—	—	82	—	—	Low
Ferreira	2002	2	2D Echo	—	—	22	—	—	Pre-KT	48	24	—	—	34	—	—	50	—	—	146	—	—	132	—	—	80	—	—	77	—	—	Very low
Montanaro	2005	2	2D Echo	—	—	22	—	—	Pre-KT	96	23	—	—	43	—	—	70	—	—	148	—	—	139	—	—	94	—	—	90	—	—	Very low
Stewart	2005	1	2D Echo	Dialysis (HD)	—	25	25	—	—	288	53	55	—	42	47	—	59	60	—	—	—	—	—	—	—	—	—	—	—	—	—	Very low
Geny	2006	1	2D Echo	—	Healthy	25	—	26	—	292	29	—	10	47	—	44	—	—	—	—	—	—	—	—	—	—	—	—	—	—	—	Very low
Hernández	2007	2	2D Echo	—	—	26	—	—	108 to 2. M pre-KT	35.7	60	—	—	51	—	—	60	—	—	—	—	—	—	—	—	—	—	—	—	—	—	Very low
Iqbal	2008	2	2D Echo	—	—	—	—	—	0 at KT	48	30	—	—	31	—	—	—	—	—	157	—	—	126	—	—	97	—	—	85	—	—	Very low
Keven	2008	3	2D Echo	Dialysis (HD)	Healthy	68	48	45	Pre-KT	48	28	23	20	40	52	—	34	36	34	—	—	—	—	—	—	—	—	—	—	—	—	Moderate
Ozkurt	2011	1	2D Echo	Dialysis (waitlist)	Healthy	24	24	25	—	≥48	22	12	12	36	45	44	64	50	33	—	—	—	—	—	—	—	—	—	—	—	—	Very low
de Souza	2012	2	2D Echo			22	—	—	Pre-KT	24	40	—	—	32	—	—	60	—	—	129	—	—	115	—	—	85	—	—	74	—	—	Low
Rocha	2012	3	2D Echo	Dialysis (waitlist)	—	—	—	—	≥48 to 2. M pre-KT	132	16	63	—	48	57	—	44	60	—	132	—	—	—	—	—	—	—	—	—	—	—	Moderate
Vaidya	2012	2	2D Echo			—	—	—	≥24 pre-KT	105.6	105	—	—	54	—	—	55	—	—	—	—	—	—	—	—	—	—	—	—	—	—	Very low
Deng	2013	2	2D Echo	—	—	—	—	—	Pre-KT	24	48	—	—	52	—	—	80	—	—	144	—	—	122	—	—	79	—	—	73	—	—	Very low
Salerno	2013	2	2D Echo	—	—	—	—	—	Pre-KT	144	104	—	—	48	—	—	63	—	—	133	—	—	—	—	—	79	—	—	—	—	—	Very low
Dounousi	2014	2	2D Echo	—	—	—	—	—	Pre-KT	≥24	12	—	—	50	—	—	75	—	—	—	—	—	—	—	—	—	—	—	—	—	—	Very low
Poulikakos	2014		2D Echo	—	Healthy	24	—	25	—	224	13	—	29	52	—	51	—	—	—	—	—	—	—	—	—	—	—	—	—	—	—	Very low
An	2015	2	2D Echo	—	—		—	—	Pre-KT	48	767	—	—	45	—	—	60	—	—	—	—	—	—	—	—	—	—	—	—	—	—	Low
Hawwa	2015	2	2D Echo	—	—	28	—	—	37 pre-KT	60,3	232	—	—	54	—	—	63	—	—	136	—	—	132	—	—	76	—	—	74	—	—	Very Low
Çolak	2015	1	2D Echo	Dialysis (HD)	Healthy	25	25	26	—	≥48	45	43	36	41	45	42	53	53	53	—	—	—	—	—	—	—	—	—	—	—	—	Very Low
Hewing	2016	2	2D Echo	—	—	25	—	—	≤30 pre-KT	76	31	—	—	44	—	—	65	—	—	140	—	—	131	—	—	82	—	—	84	—	—	Very Low
Koo	2017	2	2D Echo			24	—	—	Pre-KT	48	128			43	—	—	66	—	—	136	—	—	126	—	—	85	—	—	78	—	—	Very Low
Fujii	2019	2	2D Echo	—	—		—	—	Pre-KT	48	43	—	—	47	—	—	72	—	—	130	—	—	124	—	—	74	—	—	72	—	—	Low
Ravera	2019	1	2D Echo	Dialysis (HD)	Healthy	24	25	25	—	>96	25	25	60	52	58	53	68	64	48	—	—	—	—	—	—	—	—	—	—	—	—	Very low
Temimović	2019	1	2D Echo	—	Healthy		—	—	—	274	36	—	44	55.9			—	—	—	—	—	—	—	—	—	—	—	—	—	—	—	Very low
Kobayashi	2020	2	2D Echo			25	—	—	Pre-KT	45.8	56	—	—	48	—		73	—	—	—	—	—	—	—	—	—	—	—	—	—	—	Low
Lim	2020	3	2D Echo	Dialysis (waitlist)	—	57	62	—	Pre-KT (non-D)	48	81	85	—	33	40	—	43	50	—	136	133	—	137	135	—	82	79	—	81	81	—	Moderate
Sreedharan	2020	2	2D Echo			22	—	—	Pre-KT	48	45	—	—	34	—	—	69	—	—	154	—	—	130	—	—	92	—	—	80	—	—	Very low
Jhinger	2021	2	2D Echo				—	—	Pre-KT	24	30	—	—	34	—	—	93	—	—	—	—	—	—	—	—	—	—	—	—	—	—	Very low
Lasson	2021	2	2D Echo			26	—	—	0 at KT	24	33	—	—	47	—	—	42	—	—	136	—	—	128	—	—	75	—	—	71	—	—	Low
d’Hervé	2023	2	2D Echo	—	—	26	—	—	34.4 pre-KT	21.9	106	—	—	48	—	—	73	—	—	—	—	—	—	—	—	—	—	—	—	—	—	Moderate
Kim	2023	2	2D Echo			23	—	—	≤12 pre-KT	154	487	—	—	53	—	—	58	—	—	—	—	—	—	—	—	—	—	—	—	—	—	Low
Tat Dat	2023	2	2D Echo			20	—	—	Pre-KT	48	47	—	—	37	—	—	66	—	—	135	—	—	120	—	—	86	—	—	74	—	—	Very low
Perseghin	2005	1	CMR	Dialysis	Healthy	23	23	24	—	171	38	16	13	44	42	42	68	81	85	—	—	—	—	—	—	—	—	—	—	—	—	Very low
Patel	2008	3	CMR	Dialysis (waitlist)	—	26	26	—	Pre-KT	85.4	25	25	—	46	53	—	80	63	—	135	139	—	147	145	—	78	81	—	81	77	—	Moderate
Prasad	2018	3	CMR	Dialysis (waitlist)	—	27	30	—	Pre-KT	48	39	43	—	47	56	—	69	72	—	129	130	—	125	134	—	81	78	—	78	78	—	Moderate
Barbosa	2021	2	CMR	**—**	—	26	—	—	1 post-KT	24	44	**—**	—	50	**—**	**—**	61	—	—	139	—	—	124	—	—	80	—	—	74	—	—	Low
Qi	2022	2	CMR	—	Healthy	69	—	67	Pre-KT	58	16	—	21	21	—	—	28	—	28	—	—	—	—	—	—	—	—	—	—	—	—	Very low

Study type 1: cross-sectional study, 2 longitudinal study comparing the same KT patients before and after transplantation, and 3 studies comparing 2 groups of patients longitudinally: one transplanted and one staying on dialysis.

2D Echo, 2-dimentional echocardiography; CMR, cardiovascular magnetic resonance; D, dialysis; GRADE, grading of recommendations, assessments, development and evaluation; HD, hemodialysis; KT, kidney transplantation; M, measurement.

The evaluation of risk of bias is showed in Table S2 (**SDC**, http://links.lww.com/TXD/A657) and the assessment of certainty of evidence using grading of recommendations, assessments, development and evaluation is summarized in Table 1. Most of the evidence exhibited low quality, primarily because of the absence of a control group, randomization and blinding procedures.

### Change in LVMI in Patients Before and After KT

**FIGURE 2.**
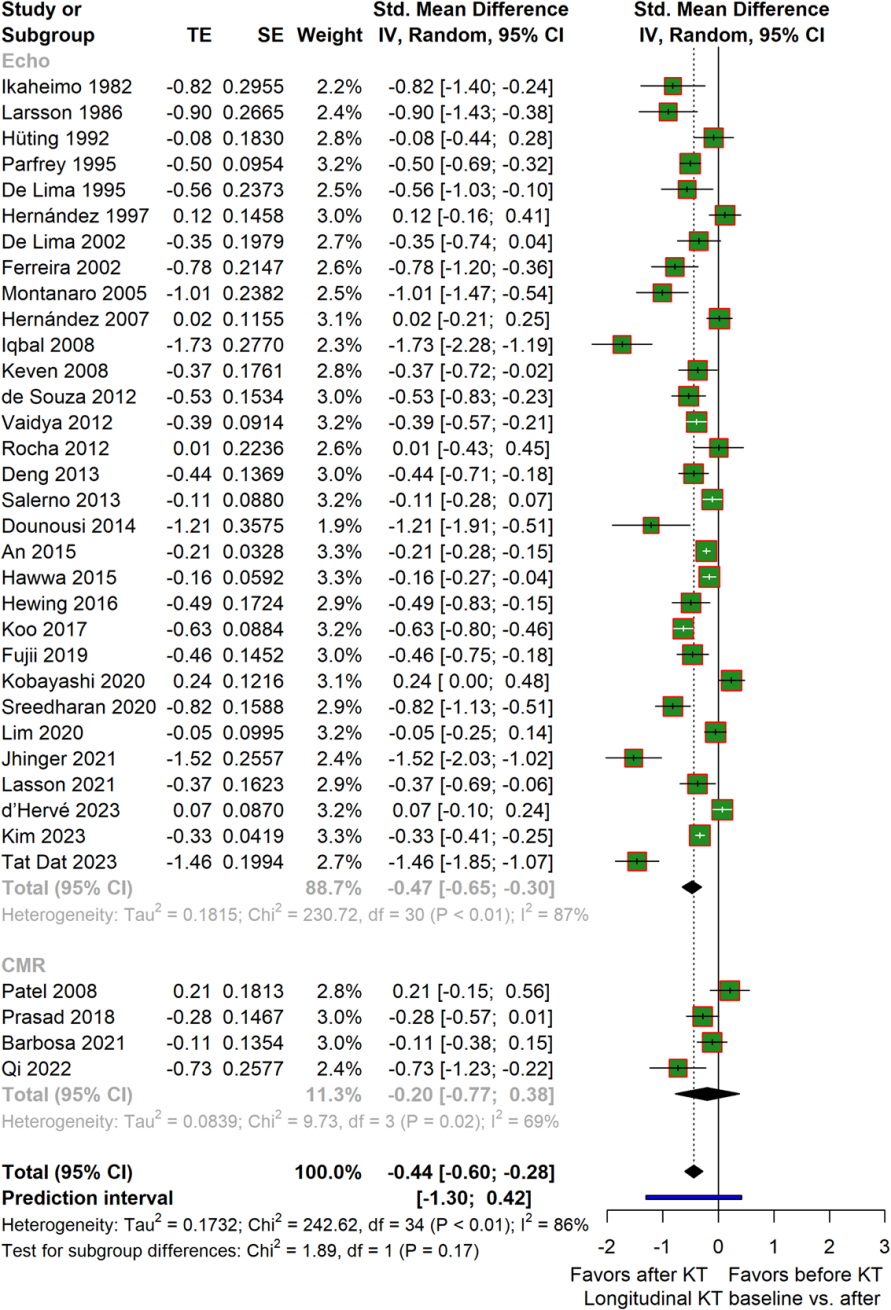
Forest plot depicting the changes in LVMI in patients before and after KT. Estimated effect sizes for LVMI changes before and after KT are presented as standardized mean difference and 95% CI. Heterogeneity analysis using *I*^2^ and Tau2 is illustrated. CI, confidence interval; KT, kidney transplantation; LVMI, left ventricular mass index; TE, total effect.

We first analyzed LVMI changes in renal recipients before and after transplantation. The outcome data were subdivided into 2 subgroups based on the imaging technique used: echocardiography or cardiovascular magnetic resonance (CMR). As shown in Figure 2, there is a significant decrease of LVMI after KT with an SMD (95% confidence interval) of −0.44 g/m^2^ (−0.60 to −0.28). Heterogeneity of these studies is high (*I*^2^ = 86%). When considering imaging modality, the effect of KT on LVM, measured with CMR, is not significant with −0.20 g/m^2^ (−0.77 to 0.38) (*I*^2^ = 69%).

To further analyze the between-study heterogeneity, several outlying and influential studies were identified (**Figure S1, SDC**, http://links.lww.com/TXD/A657). After excluding them, we performed a sensitivity analysis to evaluate the robustness of our results. As summarized in Figure S2 (**SDC**, http://links.lww.com/TXD/A657), there is still a significant reduction of LVMI after KT, with moderate shrinkage of heterogeneity. In addition, re-evaluating these influential studies by analyzing the manuscripts beyond the extracted data did not reveal any systematic difference compared with the other studies.

**FIGURE 3.**
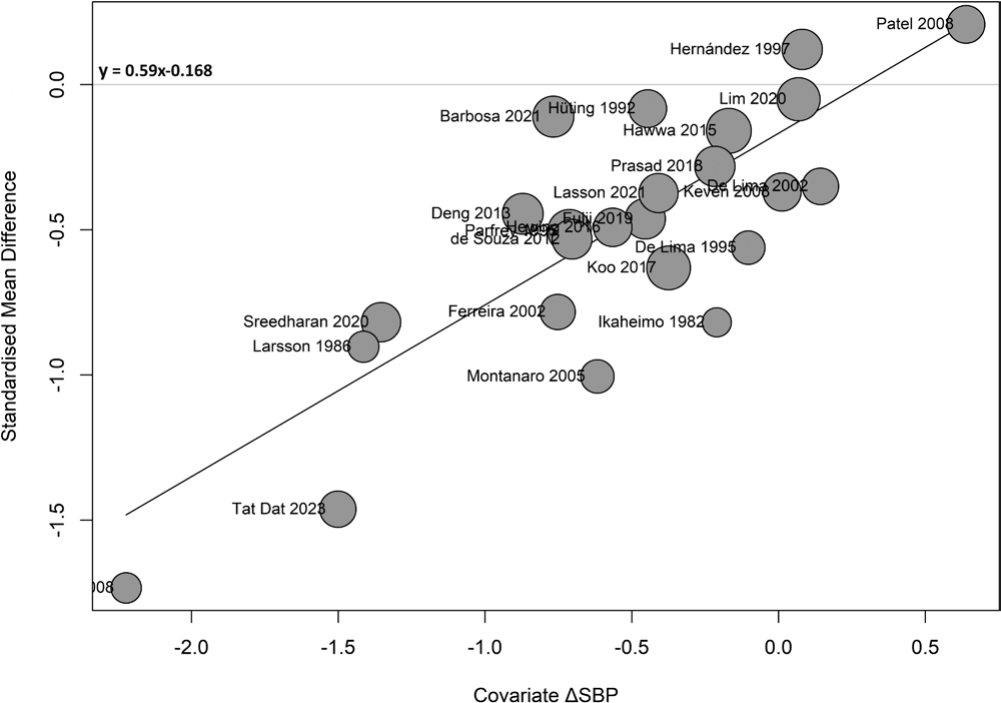
Meta-regression analysis using changes in SBP as covariate. Meta-regression analysis suggests that the reduction of SBP is a significant predictor for the decline of LVMI. The x-axis represents change in systolic pressure (mm Hg), and the y-axis represents change in LVMI as standardized mean difference. △SBP, standardized mean difference in systolic blood pressure (mm Hg) between baseline and the second measurement; LVMI, left ventricular mass index.

We also performed meta-regression of the LVMI changes on various covariates (Table 2). Changes in systolic and diastolic blood pressure during the studies are the most influential factors associated with changes in LVMI reaching *R*^2^ of 79% and 65%, respectively. Simultaneously, they can also explain a substantial amount of the differences in true effect sizes (residual heterogeneity) as well. Achieving a reduction in systolic blood pressure may be indicative of a larger decrease of LVMI (Figure 3). A comparable impact can also be observed for diastolic blood pressure (**Figure S3, SDC**, http://links.lww.com/TXD/A657). In addition, changes in hemoglobin during the studies and the age of renal recipients also influence changes in LVMI (**Figures S4 and S5, SDC**, http://links.lww.com/TXD/A657). Furthermore, dialysis duration before transplantation may also influence changes in LVMI.

**TABLE 2.** Meta-regression analysis of covariates potentially influencing left ventricular mass index

Covariate	Estimate	SE	*P*	*R*^2^, %	Residual *I*^2^, %
SBP change	0.5912	0.0931	<0.0001	79.16	61.08
DBP change	0.5569	0.1178	0.0001	64.99	72.73
SBP BL	−0.0176	0.008	0.0389	16.30	87.51
Hemoglobin change	−0.2874	0.0957	0.0076	31.88	85.78
Age	0.0326	0.0086	0.0006	36.79	90.04
Dialysis duration	0.0113	0.0053	0.047	20.47	93.62
Duration of 2. Echo to KT	0.0017	0.0018	0.3664	0.00	93.12

BL, baseline; DBP, diastolic blood pressure; KT, kidney transplantation; SBP, systolic blood pressure.

Publication bias checked by funnel plot reveals a considerable asymmetrical pattern (**Figure S6, SDC**, http://links.lww.com/TXD/A657). To ensure a symmetric funnel plot, we then calculated a bias-corrected estimate of the true effect size by imputing “missing effects” for overall and sensitivity analysis. The decrease of LVMI remains significant after KT with −0.24 g/m^2^ (−0.44 to −0.05) for the overall analysis and −0.39 g/m^2^ (−0.52 to −0.26) for the sensitivity analysis, whereas the funnel plots exhibit symmetry (**Figures S7–S10, SDC**, http://links.lww.com/TXD/A657).

### Change in LVMI in Renal Transplant Recipients and Patients Remaining on Dialysis

**FIGURE 4.**
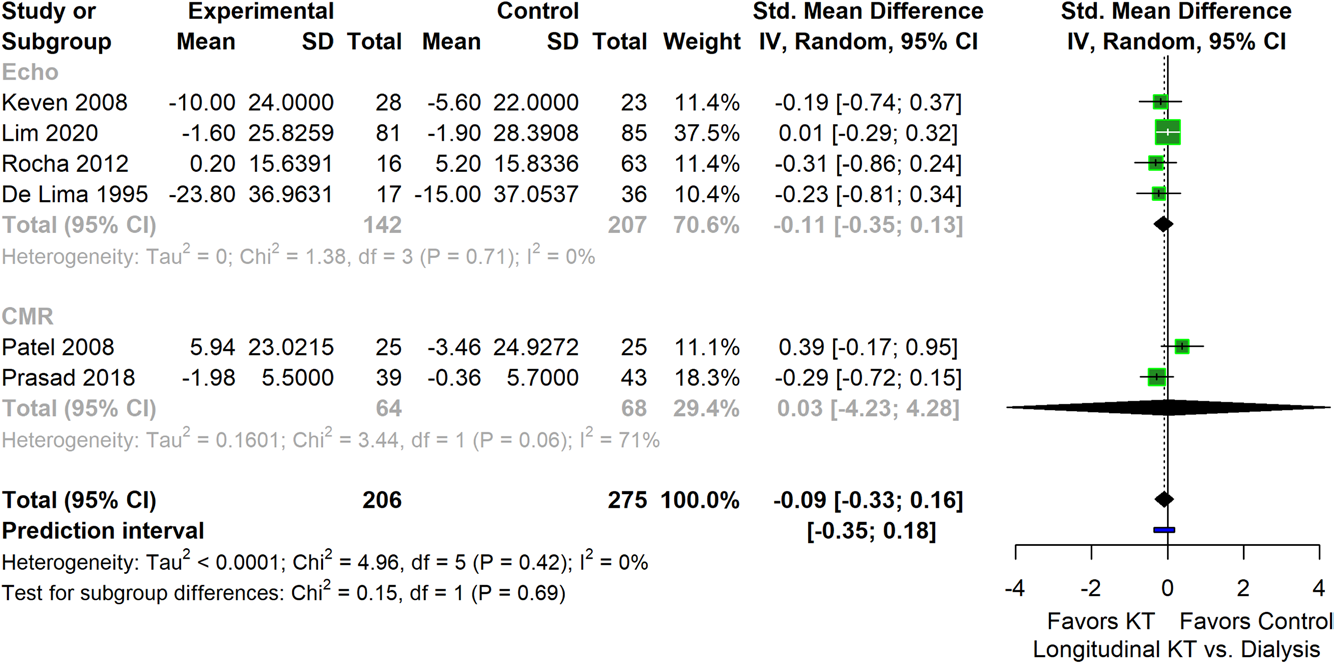
Forest plot depicting the changes in LVMI in renal recipients compared with patients remaining on dialysis. Estimated effect sizes for LVMI changes are presented as standardized mean difference and 95% CI. Heterogeneity analysis using *I*^2^ and Tau2 is illustrated. CI, confidence interval; KT, kidney transplantation; LVMI, left ventricular mass index.

Six longitudinal studies reported LVMI in renal transplant recipients before and after transplantation while including participants remaining on dialysis with comparable baseline characteristics as control group. Compared with the dialysis group, no significant difference in change of LVMI assessed from baseline to follow-up was observed in transplantation group (−0.09 g/m^2^ [−0.33 to 0.16]) without heterogeneity (*I*^2^ = 0%) (Figure 4).

**FIGURE 5.**
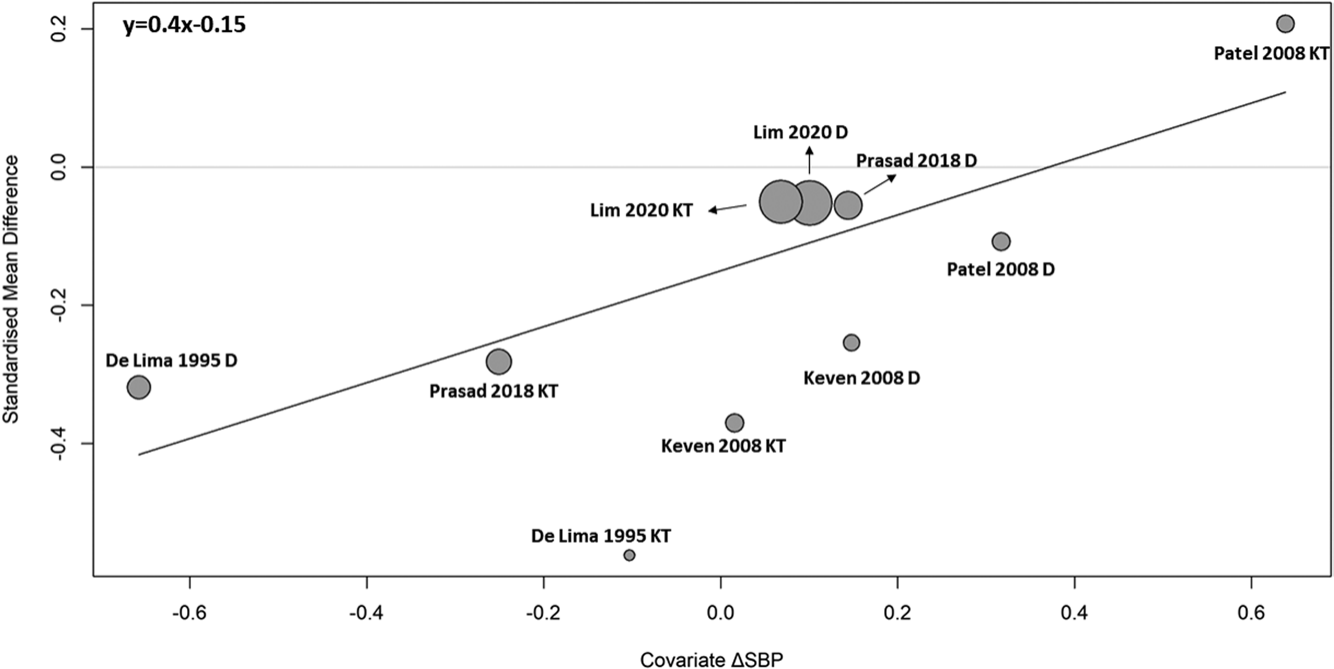
Meta-regression analysis for kidney transplantation vs dialysis separated using changes in SBP as a covariate. To evaluate the impact of blood pressure control on LVMI, we separated the KT and control (dialysis) groups of longitudinal studies with kidney recipients and patients remaining on dialysis. The reduction in SBP could serve as a predictor for the regression of left ventricular hypertrophy in both KT group and dialysis groups. The x-axis represents change in systolic pressure (mm Hg), and the y-axis represents change in LVMI as standardized mean difference. △SBP, standardized mean difference in systolic blood pressure (mm Hg) between baseline and the second measurement; D, dialysis; KT, kidney transplantation; LVMI, left ventricular mass index.

To address the inconsistency between the 2 analyses, which involved longitudinal studies with or without a control group, we further evaluated the impact of blood pressure control on LVMI by separating the transplantation and control groups. A meta-regression using change of systolic blood pressure as a covariate was then conducted (Figure 5). This reinforces that the reduction of systolic blood pressure is associated with a decrease of LVMI, irrespective of whether the patients had been subjected to KT or continued on dialysis.

### Cross-sectional Studies

LVMI as an outcome was extracted from 21 studies involving renal recipients after KT and control groups, which included dialysis-dependent and healthy individuals.

Renal transplant recipients exhibited lower LVMI compared with patients remaining on dialysis (−0.42 g/m^2^ [−0.63 to −0.21]), but showed significantly higher LVMI compared with healthy individuals (0.70 g/m^2^ [0.19 to 1.21]) (**Figures S11 and S12, SDC**, http://links.lww.com/TXD/A657).

## DISCUSSION

LVH is a frequent and well-described phenomenon associated with chronic kidney disease and end-stage kidney disease. Its prevalence and progression correlate with the deterioration of renal function.^62^ Current evidence indicates that chronic hemodialysis is associated with LVH.^63,64^ Increased LVM is closely linked to high risk for de novo congestive heart failure and cardiovascular mortality.^65^ Hence, KT, as the preferred renal replacement modality, provides an opportunity to reverse LVH. Our meta-analysis incorporated all available evidence from cross-sectional and longitudinal studies examining the impact of KT on LVMI including information from 4122 trial participants. Our results indicate a potential cardiovascular benefit, defined as the regression of LVH, after KT. This benefit was primarily attributed to improved blood pressure control rather than the transplantation itself. Our results also suggested that hemoglobin level and age could also influence LVM after KT.

LVH affects nearly half of kidney transplant recipients and stands as a dominant determinant of cardiovascular survival.^66,67^ This can be primarily explained by accelerated myocardial remodeling in response to hemodynamic stresses and exposure to uremia during the dialysis period.^67^ It was generally believed that rectifying the uremic state through KT could lead to the regression of LVH. However, evidence suggested that LVH regresses, though not entirely.^25,67,68^ Numerous risk factors related to transplant status might contribute to this discrepancy. Volume and pressure overload could still persist if renal function is not sufficiently restored after transplantation. LVH may be further promoted by hypertension, posttransplant diabetes and anemia. In addition, it is believed that immunosuppressive therapy also modulates cardiac growth through sodium and water retention because of steroid use and the induction of hypertension, hyperlipidemia, or metabolic disease by calcineurin inhibitors.^69,70^ In our study, we also observed this discrepancy. Kidney transplant recipients exhibited a significant reduction in LVMI after transplantation. However, there was high heterogeneity, and this benefit was diminished when compared with the dialysis control group.

Hypertension is prevalent in a majority of patients before transplantation and frequently persists or intensifies because of specific immunosuppressive regimens, donor age, and graft dysfunction posttransplantation.^71–73^ Optimizing blood pressure is a key element in modifying LVH in patients with chronic kidney disease and end stage renal disease.^74^ The same holds true for posttransplant patients. In a study involving kidney transplant recipients with a 4-y follow-up, the duration and severity of hypertension surfaced as predictors of failure to regress.^75^ Another randomized double-blinded trial with 154 renal transplant recipients indicated a well-controlled blood pressure is associated with a significant improvement of LVMI.^76^ In an analysis of pediatric patients, stricter blood pressure control was found to be associated with regression of LVH.^77^ The findings of our meta-analysis align with evidence from previous studies. The alteration in blood pressure is directly linked to the change in LVM, not just in kidney transplant patients but also in those remaining on dialysis. Moreover, our analysis also indicates that hypertension is an independent predictive factor for LVH after KT.

Before or at the time of KT, the cardiac geometry is influenced by a complex interplay of traditional cardiovascular risk factors, including age, diabetes, anemia, hypertension, etc.^78^ Anemia after KT is also prevalent and exhibits a biphasic pattern.^79^ It acts as a significant hemodynamic stimulus for cardiac growth. One study showed a gradual, inverse association between hemoglobin levels and the presence of LVH,^80^ which is consistent with our findings. Age is regarded as a nonmodifiable risk factor for adverse cardiovascular outcomes. Advanced age at baseline was identified as an important predictor for nonregression of LVH after transplantation.^75^ Based on our analysis, patients of advanced age undergoing KT should be subjected to more frequent monitoring and provided with more precise management with regard to blood pressure control and echocardiographic surveillance as well as strict optimization of other cardiovascular risk factors as posttransplant diabetes or hyperlipidemia.

Our meta-analysis has several limitations: the majority of studies included in our analysis demonstrate a moderate to high risk of bias, primarily attributed to the absence of a randomized blinded controlled study design and relatively small sample size. Nevertheless, carrying out a randomized blinded trial in the context of KT is nearly impossible in the real world. Second, compared with 2-dimensional echocardiography, CMR imaging is deemed more precise for evaluating left ventricular dimensions, particularly in patients with renal dysfunction.^81^ Only 5 studies evaluated LVMI using CMR imaging. Three of them indicated no difference in LVMI after KT. It is worth noting that all these studies had a small sample size, so we have to interpret this result with caution. In addition, LVH may worsen or develop de novo during the years after renal function deterioration after transplantation. To address this issue, we specifically included studies with the second measurement of LVMI occurring ≥6 mo and up to 3 y after KT. At last, considerable heterogeneity was detected in our analysis for LVMI changes before and after KT. Therefore, we performed a sensitivity analysis, excluding outlying and influential studies, as well as an analysis addressing publication bias. Both yielded outcomes comparable to the main study, supporting our main conclusion.

In conclusion, our meta-analysis of the existing longitudinal and cross-sectional studies has revealed that LVM might regress after KT, suggesting a potential cardiovascular benefit in terms of long-term survival. Yet, the observed improvement in cardiac growth can be primarily ascribed to the effective control of blood pressure during the follow-up period rather than to the renal transplant itself. In this study, we also found that ameliorating anemia status is linked to the regression of LVH, whereas age at the time of transplantation remains a nonmodifiable predictive factor. The current findings emphasize that, in addition to preventing rejection and managing infectious complications through the modification of immunosuppression, controlling blood pressure is also an important aspect of posttransplant care. Posttransplant care also should include assessment of LVM on a regular basis. Transplant physicians must be aware that transplantation itself does not necessarily lead to an improvement in LVMI reflecting cardiac risk.

## Supplementary Material


