# Supplementary material for: Left Ventricular Hypertrophy After Renal Transplantation: Systematic Review and Meta-analysis
Source: Transplant Direct. 2024 May 17;10(6):e1647. doi: 10.1097/TXD.0000000000001647 (PMC11104731; doi:10.1097/TXD.0000000000001647)
Supplement: Supplementary file 1 [file txd-10-e1647-s001.pdf]

Section/topic	#	Checklist item	Reported on page #
**TITLE**			
Title	1	Identify the report as a systematic review, meta-analysis, or both.	1
**ABSTRACT**			
Structured summary	2	Provide a structured summary including, as applicable: background; objectives; data sources; study eligibility criteria, participants, and interventions; study appraisal and synthesis methods; results; limitations; conclusions and implications of key findings; systematic review registration number.	4-5
**INTRODUCTION**			
Rationale	3	Describe the rationale for the review in the context of what is already known.	6
Objectives	4	Provide an explicit statement of questions being addressed with reference to participants, interventions, comparisons, outcomes, and study design (PICOS).	6
**METHODS**			
Protocol and registration	5	Indicate if a review protocol exists, if and where it can be accessed (e.g., Web address), and, if available, provide registration information including registration number.	7 Pragraph 1
Eligibility criteria	6	Specify study characteristics (e.g., PICOS, length of follow-up) and report characteristics (e.g., years considered, language, publication status) used as criteria for eligibility, giving rationale.	7 Paragraph 2
Information sources	7	Describe all information sources (e.g., databases with dates of coverage, contact with study authors to identify additional studies) in the search and date last searched.	7 Paragraph 3

Search	8	Present full electronic search strategy for at least one database, including any limits used, such that it could be repeated.	7 Paragraph 3
Study selection	9	State the process for selecting studies (i.e., screening, eligibility, included in systematic review, and, if applicable, included in the meta-analysis).	7 Paragraph 4
Data collection process	10	Describe method of data extraction from reports (e.g., piloted forms, independently, in duplicate) and any processes for obtaining and confirming data from investigators.	8 Paragraph 1
Data items	11	List and define all variables for which data were sought (e.g., PICOS, funding sources) and any assumptions and simplifications made.	8 Paragraph 2
Risk of bias in individual studies	12	Describe methods used for assessing risk of bias of individual studies (including specification of whether this was done at the study or outcome level), and how this information is to be used in any data synthesis.	8 Paragraph 1
Summary measures	13	State the principal summary measures (e.g., risk ratio, difference in means).	8 Paragraph 3
Synthesis of results	14	Describe the methods of handling data and combining results of studies, if done, including measures of consistency (e.g., I^2) for each meta-analysis.	9
Risk of bias across studies	15	Specify any assessment of risk of bias that may affect the cumulative evidence (e.g., publication bias, selective reporting within studies).	9
Additional analyses	16	Describe methods of additional analyses (e.g., sensitivity or subgroup analyses, meta-regression), if done, indicating which were pre-specified.	9
**RESULTS**			
Study selection	17	Give numbers of studies screened, assessed for eligibility, and included in the review, with	10 Paragraph 1<br>Figure 1

		reasons for exclusions at each stage, ideally with a flow diagram.	
Study characteristics	18	For each study, present characteristics for which data were extracted (e.g., study size, PICOS, follow-up period) and provide the citations.	10 Paragraph 1<br>27-29 Table 1
Risk of bias within studies	19	Present data on risk of bias of each study and, if available, any outcome level assessment (see item 12).	10 Paragraph 2 Supplementary Table 1
Results of individual studies	20	For all outcomes considered (benefits or harms), present, for each study: (a) simple summary data for each intervention group (b) effect estimates and confidence intervals, ideally with a forest plot.	10-12
Synthesis of results	21	Present results of each meta-analysis done, including confidence intervals and measures of consistency.	10-12
Risk of bias across studies	22	Present results of any assessment of risk of bias across studies (see Item 15).	10-11 Supplementary Material
Additional analysis	23	Give results of additional analyses, if done (e.g., sensitivity or subgroup analyses, meta-regression [see Item 16]).	11-12
**DISCUSSION**			
Summary of evidence	24	Summarize the main findings including the strength of evidence for each main outcome; consider their relevance to key groups (e.g., healthcare providers, users, and policy makers).	13-15
Limitations	25	Discuss limitations at study and outcome level (e.g., risk of bias), and at review-level (e.g., incomplete retrieval of identified research, reporting bias).	15-16
Conclusions	26	Provide a general interpretation of the results in the context of other evidence, and implications for future research.	17

FUNDING			
Funding	27	Describe sources of funding for the systematic review and other support (e.g., supply of data); role of funders for the systematic review.	2

Table S1. PRISMA-Checklist of Items to Include when reporting a Systematic Review

	D1	D2	D3	D4	D5	D6	D7	D8	Overall
An 2015									Moderate to High
Barbosa 2021									Moderate
Çolak 2015									High
Covic 1998									High
d'Hervé 2023									Low
De Lima 1999									High
De Lima 2002									Moderate
De Lima 1995									Low
de Souza 2012									Moderate
Deng 2013									Moderate to High
Dounousi 2014									Moderate
Ferreira 2002									Moderate to High
Fujii 2019									Moderate
Geny 2006									High
Hawwa 2015									High
Hernández 2007									High
Hernández 1997									Moderate

**D1**	Was selection of exposed and no-exposed cohorts drawn from the same population?
**D2**	Can we be confident in the assessment of exposure?
**D3**	Can we be confident that the outcome of interest was not present at start of study?
**D4**	Did the study match exposed and unexposed for all variables that are associated with the outcome of interest or did the statistical analysis adjust for these prognostic variables?
**D5**	Can we be confident in the assessment of the presence or absence of the prognostic factors?
**D6**	Can we confident in the assessment of outcome?

Iqbal 2008									High
Jhinger 2021									Moderate to High
Keven 2008									Low
Kim 2023									Moderate to High
Kaboyashi 2020									Moderate
Koo 2017									Moderate to High
Larsson 1986									High
Lassen 2021									Moderate
Lim 2020									Low
Montanaro 2005									High
Ozkurt 2011									High
Parfrey 1995									Moderate to High
Patel 2008									Low
Perseghin 2005									Moderate to High
Poulikakos 2014									Moderate to High
Prasad 2018									Low
Qi 2022									Moderate to High
Ravera 2019									High
Rocha 2012									Low
Salerno 2013									Moderate to High
Sreedharan 2020									High

**D8**	Were co-interventions similar between groups?

definitely yes

probably yes/probably no

definitely no

Stewart 2005									Moderate to High
Suwelack 1999									Moderate to High
Tat Dat 2023									High
Temimović 2019									High
Vaidya 2012									Moderate to High

Table S2. Risk of Bias

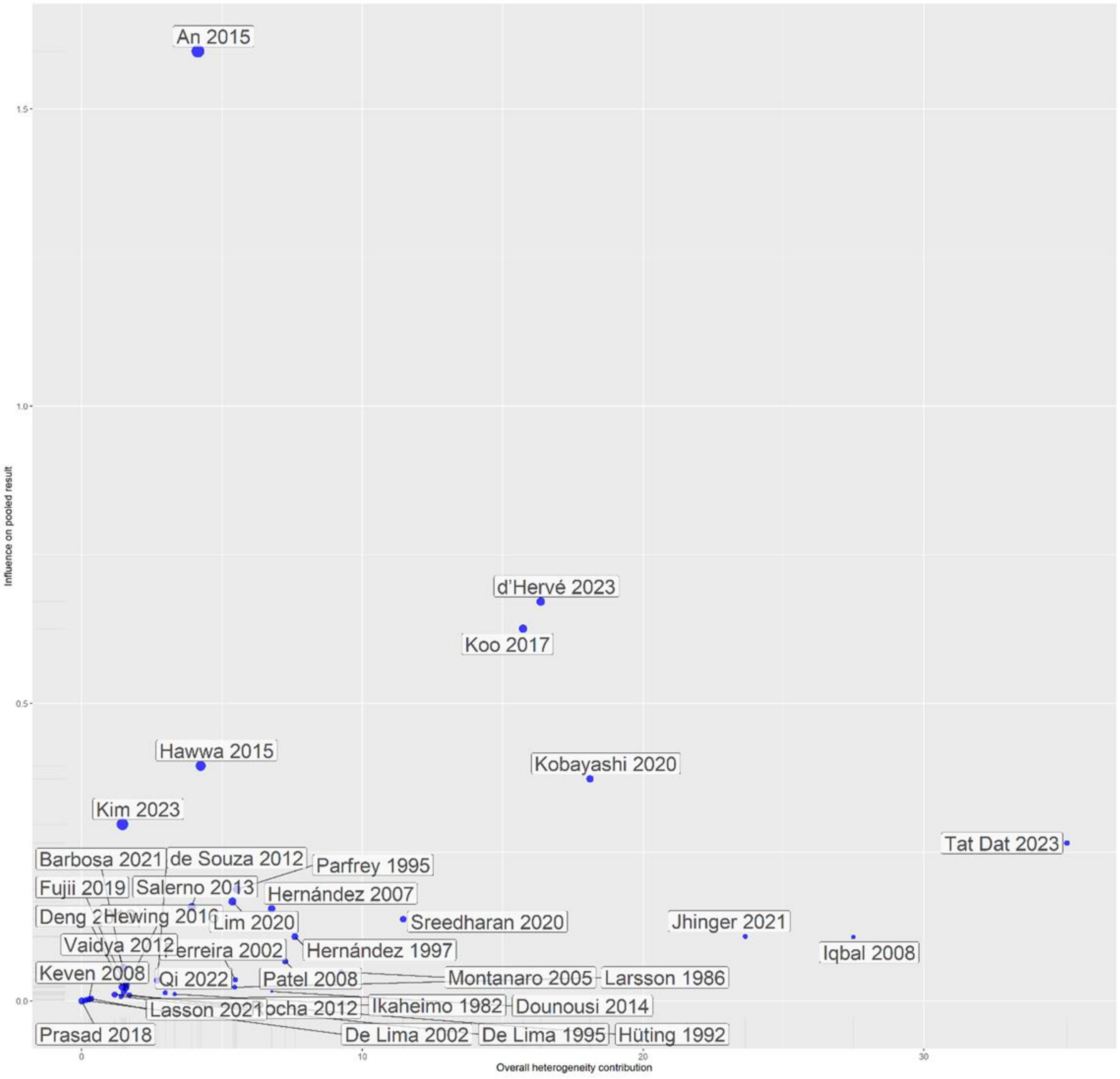

Figure S1. Influential analysis

The influential analysis is outlined using Baujat plot. The x-axis shows the overall heterogeneity contribution of each effect size while the y-axis shows the influence of each effect size on the pooled result. Studies with high x-axis value and/or with high y-axis value could be considered as influential cases. Studies in the upper right corner of the plot may be most influential, as they have a substantial impact on both the estimated heterogeneity and the pooled effect. Therefore, studies from An et al. 2015, d'Hervé et al. 2023, Kobayashi et al. 2020, Jhinger et al. 2021, Iqbal 2008 and Tat Dat 2023 would be considered as influential.

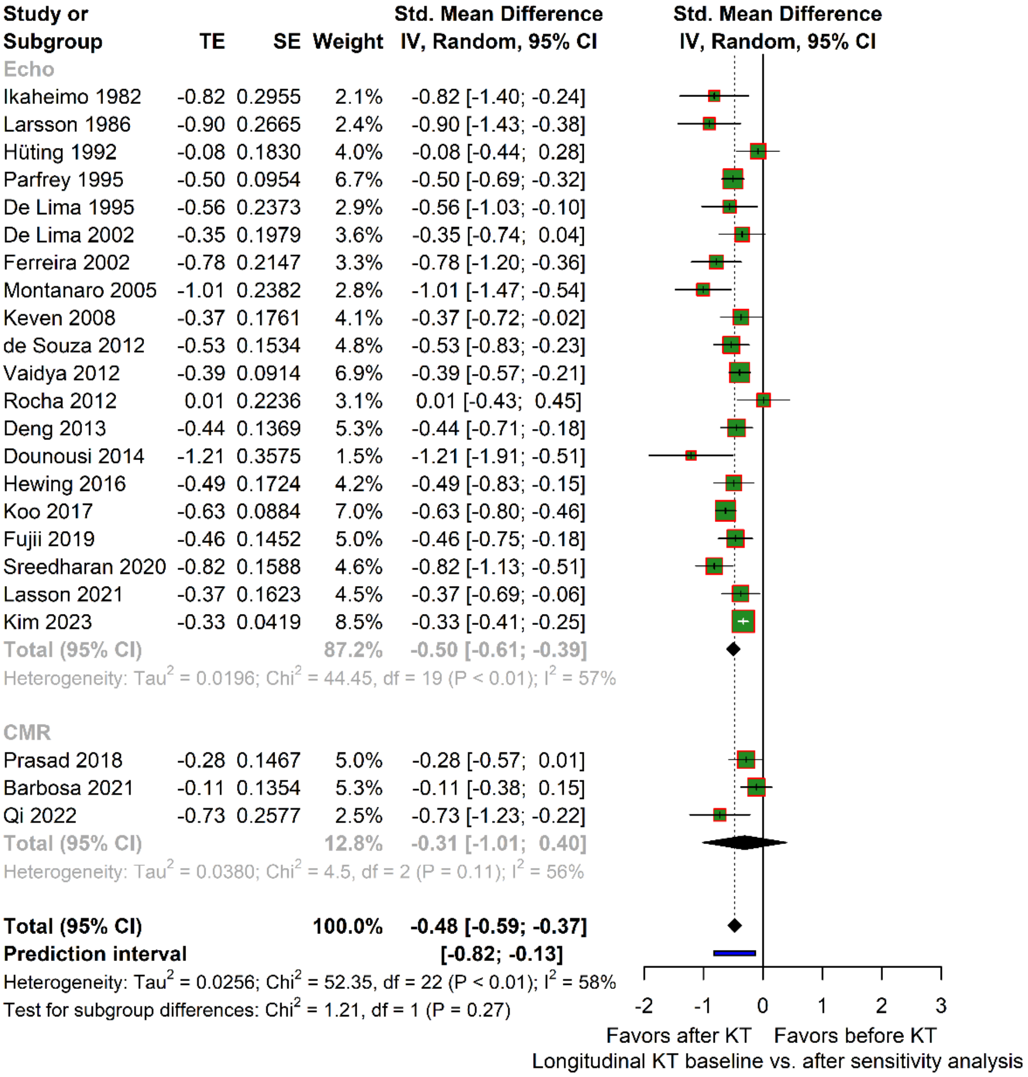

Figure S2. Forest plot of change in LVMI before and after kidney transplantation sensitivity analysis.

After removing outlying and influential studies, a sensitivity analysis was conducted for longitudinal studies before and after kidney transplantation without control group. Estimated effect sizes are presented as standardized mean difference and 95% confidence interval. Heterogeneity analysis using I^2 and Tau^2 is illustrated.

KT: kidney transplantation.

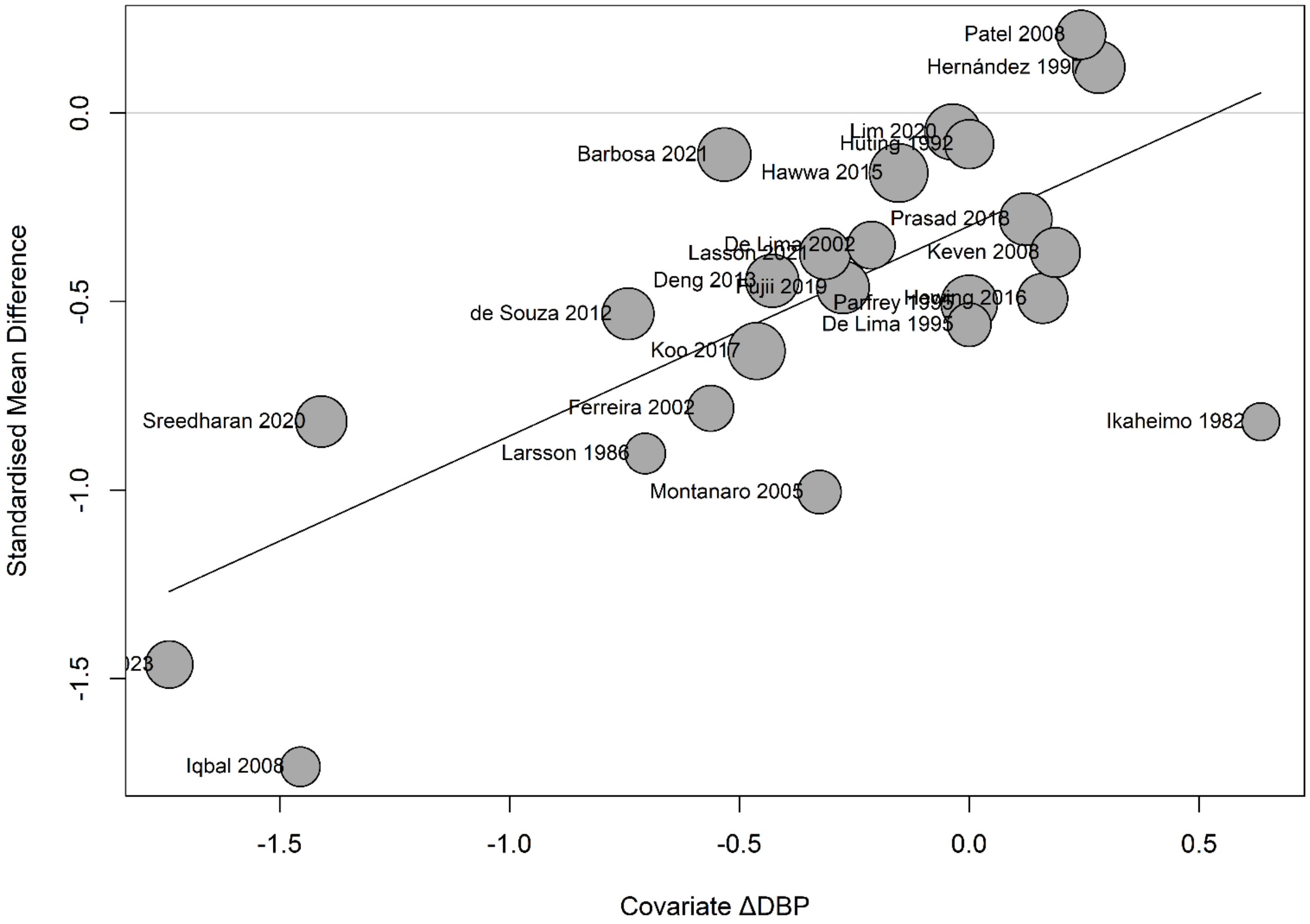

Figure S3. Meta-regression analysis using changes in diastolic blood pressure as covariate

X-axis represents change in diastolic pressure (mmHg), y-axis represents change in LVMI as standardized mean difference.

△DBP, standardized mean difference in diastolic blood pressure (mmHg) between baseline and the second measurement

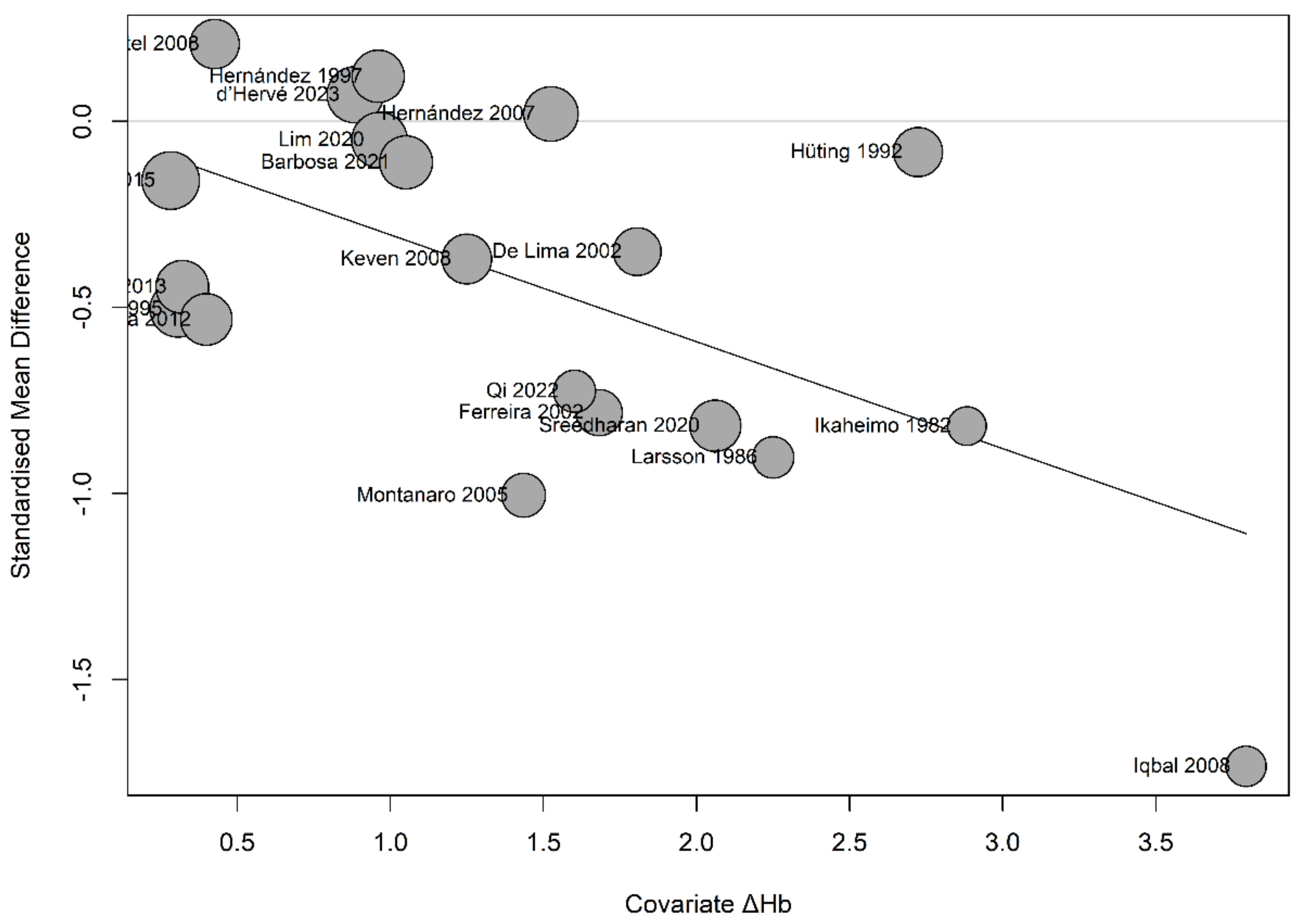

Figure S4. Meta-regression analysis using change in hemoglobin as covariate

X-axis represents change in hemoglobin (g/dl), y-axis represents change in LVMI as standardized mean difference.

△Hb, standardized mean difference in hemoglobin (g/dl) between baseline and the second measurement.

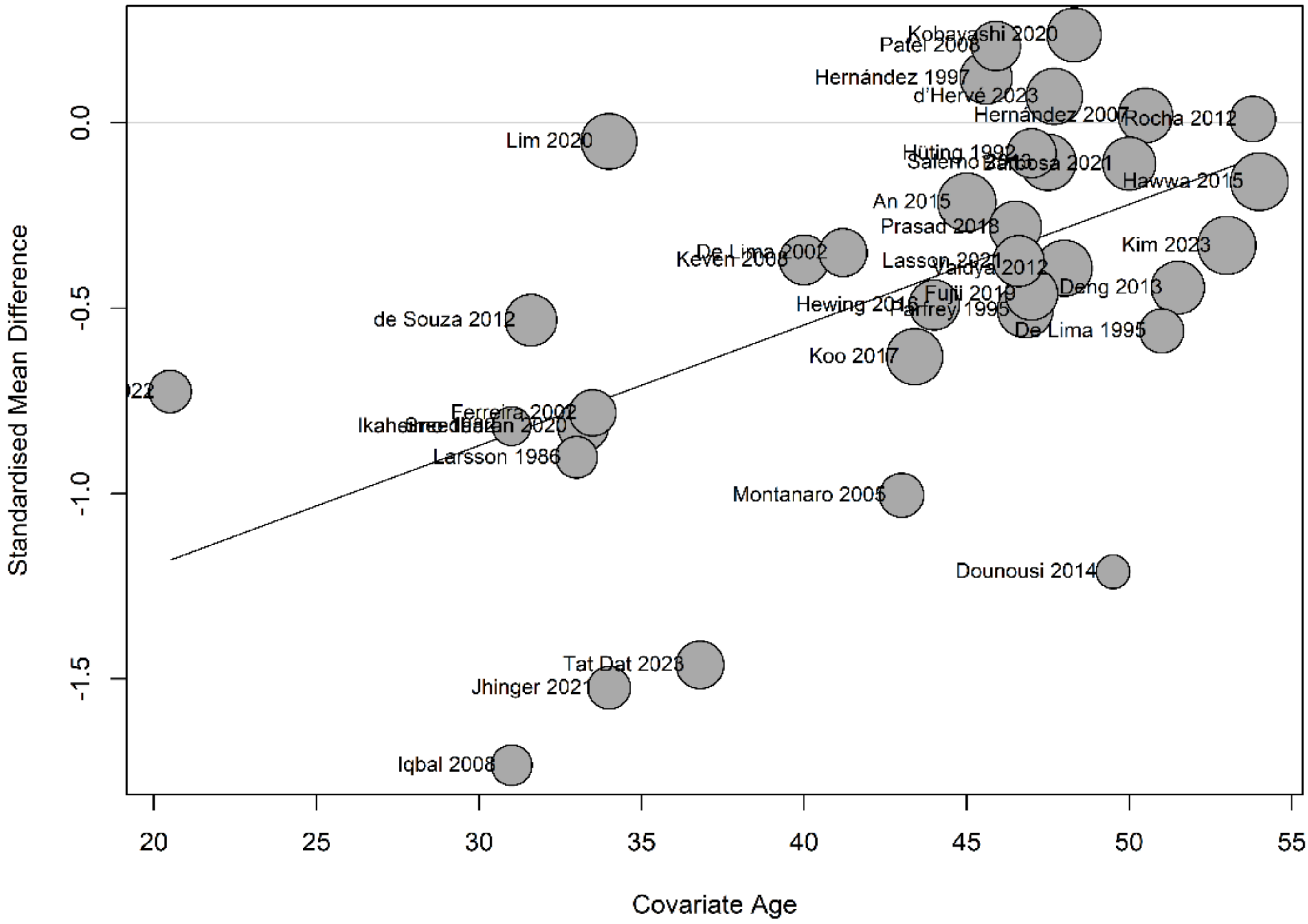

Figure S5. Meta-regression analysis using age at baseline as covariate

X-axis represents age at baseline (years), y-axis represents change in LVMI as standardized mean difference.

Age, mean age at baseline.

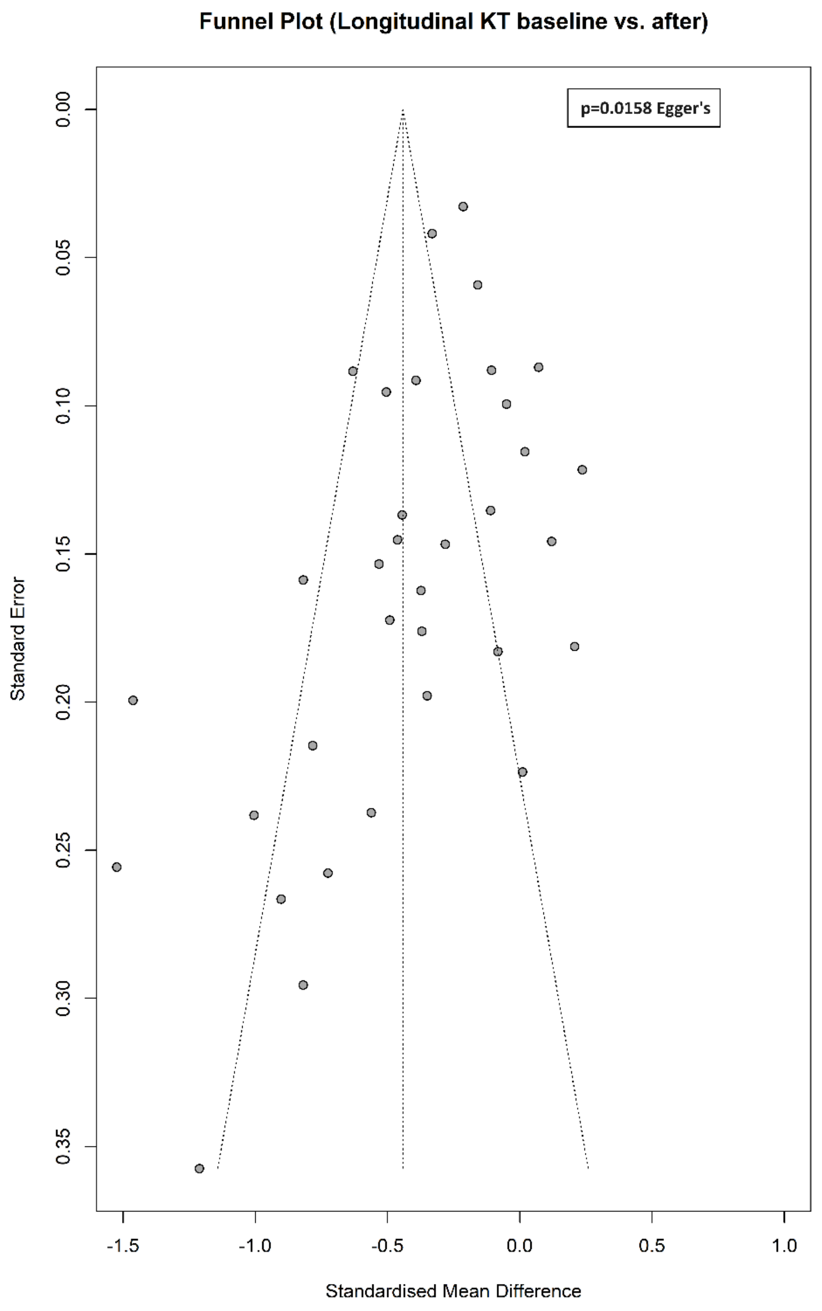

Figure S6. Funnel plot of publication bias

Funnel plot indicates a considerable publication bias in studies examining changes of LVMI before and after kidney transplantation.

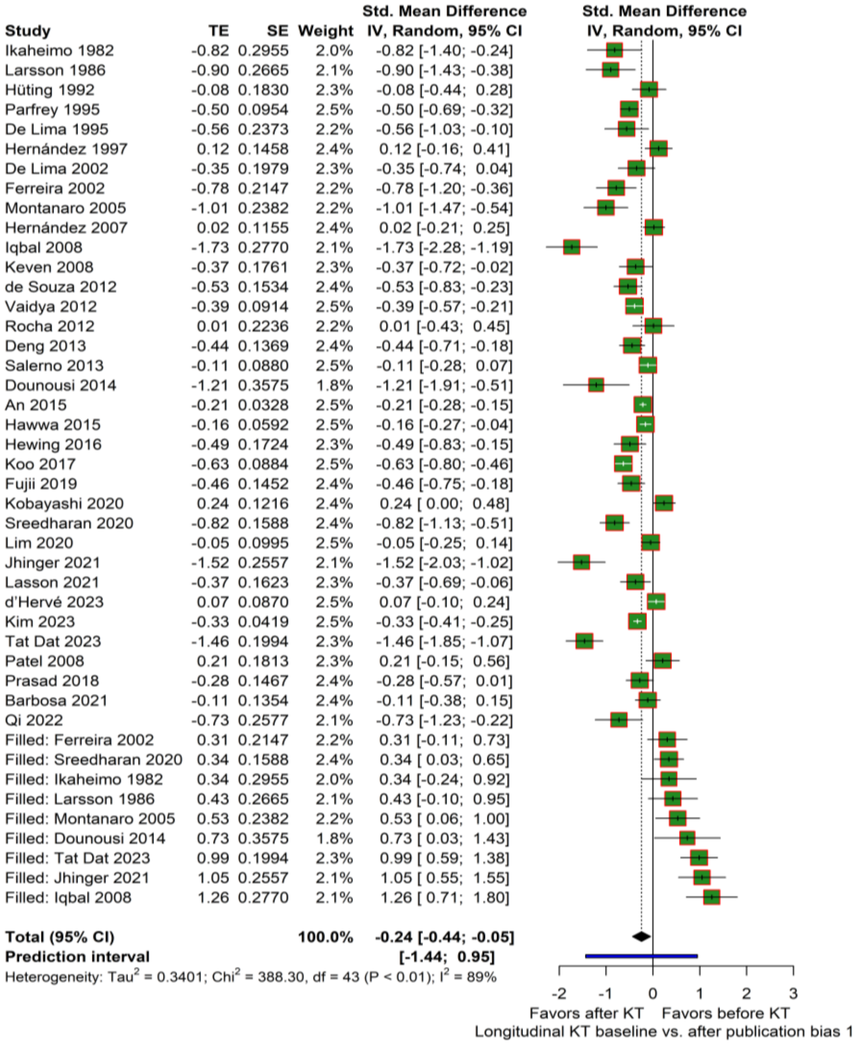

Figure S7. Forest plot for all studies using Duval & Tweedie trim and fill method

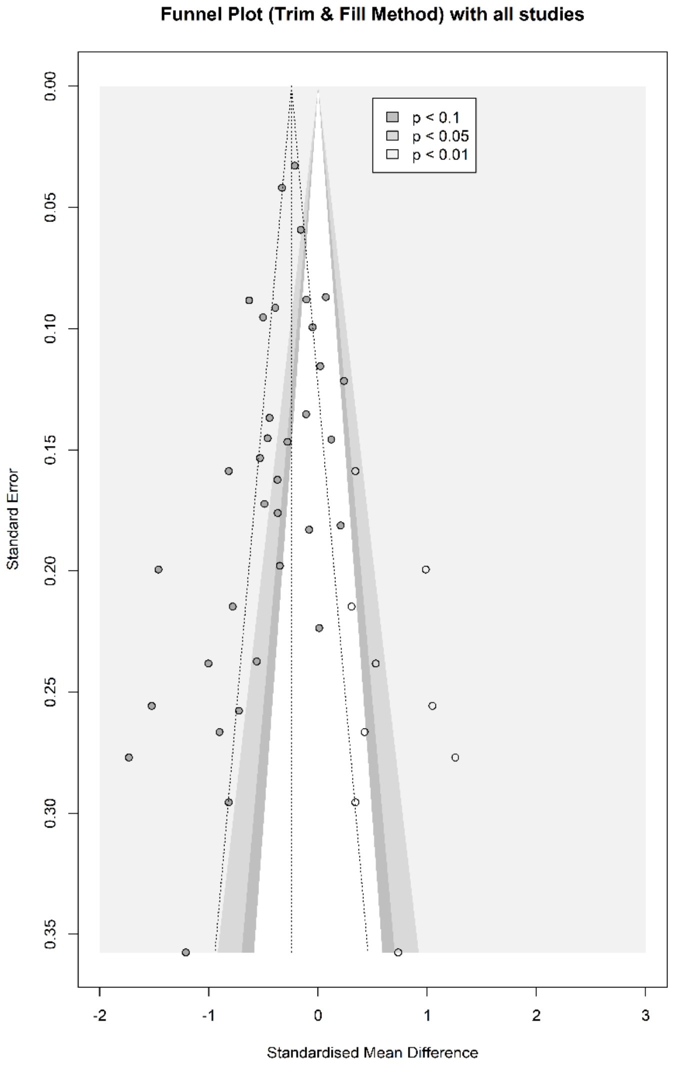

Figure S8. Funnel plot for all studies using Duval & Tweedie trim and fill method

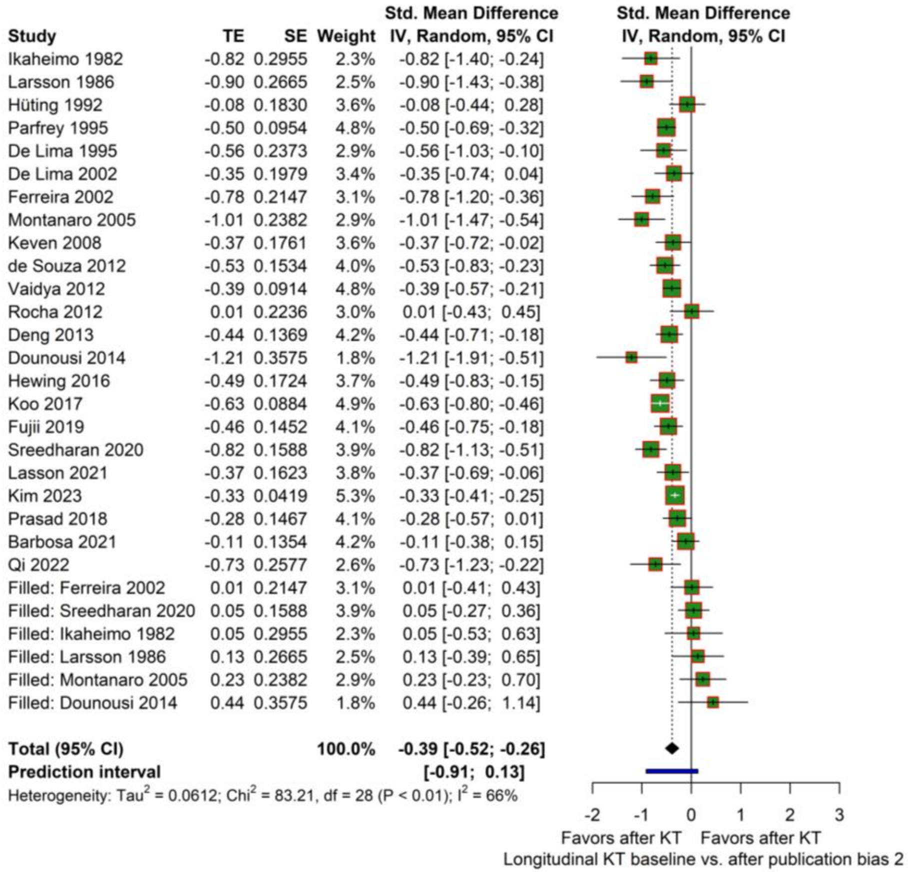

Figure S9. Forest plot of sensitivity analysis using Duval & Tweedie trim and fill method

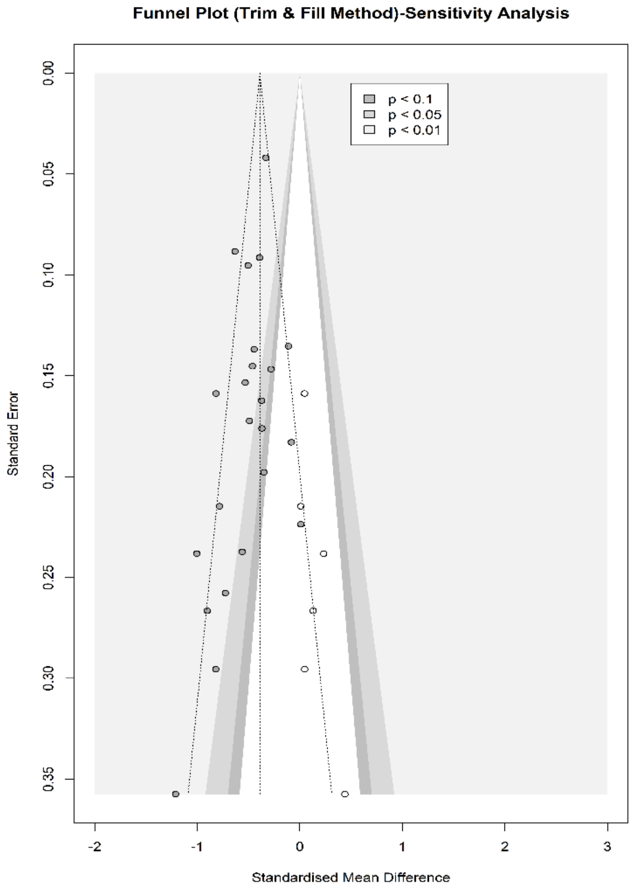

Figure S10. Funnel plot for all studies using Duval & Tweedie trim and fill method

To explore the extent to which publication bias may distort our estimate, we employed the trim and fill method to impute "missing" effects until the funnel plot achieved symmetry. Considering the significant heterogeneity in our analysis, we also conducted a sensitivity analysis after excluding all outlying and influential studies. The imputed studies are represented by circles that have no fill color in the funnel plots. The impact of kidney transplantation on the regression of LVMI stays significant.

Study or Subgroup	Experimental Mean	Experimental SD	Experimental Total	Control Mean	Control SD	Control Total	Weight	Std. Mean Difference IV, Random, 95% CI
**Echo**								
De Lima 1995	132.90	31.0000	16	155.60	43.1000	36	6.0%	-0.56 [-1.16; 0.04]
Covic 1998	173.10	48.0000	28	185.10	81.9000	35	7.7%	-0.17 [-0.67; 0.33]
De Lima 1999	141.00	38.0000	31	164.00	54.0000	73	9.2%	-0.46 [-0.88; -0.03]
Stewart 2005	145.50	43.0000	53	161.80	69.5000	55	10.4%	-0.28 [-0.66; 0.10]
Keven 2008	119.00	32.0000	28	145.00	38.0000	23	6.4%	-0.74 [-1.31; -0.16]
Ozkurt 2011	110.60	25.8000	22	142.40	30.7000	12	4.2%	-1.13 [-1.88; -0.37]
Rocha 2012	57.50	18.3000	16	60.50	21.9000	63	6.7%	-0.14 [-0.69; 0.41]
Çolak 2015	65.60	14.5000	45	75.20	20.9000	43	9.2%	-0.53 [-0.96; -0.11]
Ravera 2019	90.00	29.0000	25	126.00	47.0000	25	6.2%	-0.91 [-1.49; -0.32]
Lim 2020	103.30	26.8000	81	111.90	35.4000	85	12.6%	-0.27 [-0.58; 0.03]
**Total (95% CI)**			**345**			**450**	**78.6%**	**-0.43 [-0.62; -0.24]**
Heterogeneity: $Tau^2 = 0.0005$; $Chi^2 = 11.05$, df = 9 (P = 0.27); $I^2 = 19\%$								
**CMR**								
Patel 2008	99.40	32.1000	25	89.80	33.6000	25	6.6%	0.29 [-0.27; 0.85]
Perseghin 2005	77.90	15.6000	38	96.50	24.1000	16	5.7%	-0.99 [-1.61; -0.38]
Prasad 2018	27.80	5.5000	39	29.70	9.3000	43	9.0%	-0.24 [-0.68; 0.19]
**Total (95% CI)**			**102**			**84**	**21.4%**	**-0.30 [-1.87; 1.26]**
Heterogeneity: $Tau^2 = 0.3082$; $Chi^2 = 9.16$, df = 2 (P = 0.01); $I^2 = 78\%$								
**Total (95% CI)**			**447**			**534**	**100.0%**	**-0.42 [-0.63; -0.21]**
**Prediction interval**								**[-0.89; 0.05]**
Heterogeneity: $Tau^2 = 0.0378$; $Chi^2 = 21.09$, df = 12 (P = 0.05); $I^2 = 43\%$								
Test for subgroup differences: $Chi^2 = 0.11$, df = 1 (P = 0.74)								

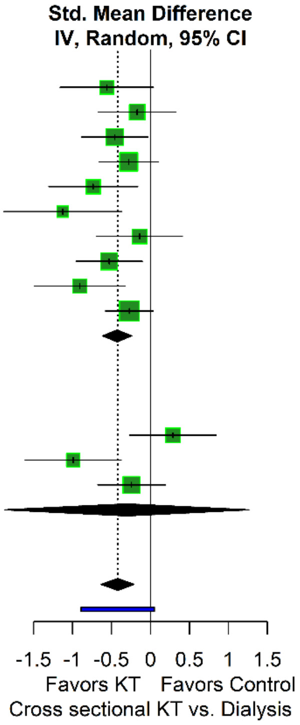

Figure S11. Forest plot illustrating cross-sectional studies with renal transplant recipients and dialysis patients

Estimated effect sizes for LVMI changes are presented as standardized mean difference and 95% confidence interval. Heterogeneity analysis using I^2 and Tau^2 is illustrated. Overall renal recipients exhibited milder left ventricular hypertrophy compared to patients remaining on dialysis (-0.42 g/m2 [-0.63; -0.21]; P = 0.05) with moderate heterogeneity.

KT: kidney transplantation.

Study or Subgroup	Experimental Mean	SD	Total	Control Mean	SD	Total	Weight	Std. Mean Difference IV, Random, 95% CI
Echo								
Suwelack 1999	146.00	41.4000	35	83.00	21.5000	29	10.2%	1.84 [1.24; 2.43]
Geny 2006	109.30	32.3000	19	96.00	23.4000	10	9.0%	0.44 [-0.34; 1.21]
Ozkurt 2011	110.60	25.8000	22	100.84	22.3900	12	9.4%	0.39 [-0.32; 1.10]
Poulikakos 2014	138.90	31.0000	13	105.30	26.2000	29	9.5%	1.19 [0.48; 1.90]
Çolak 2015	65.60	14.5000	45	62.00	14.6000	36	11.1%	0.25 [-0.19; 0.69]
Ravera 2019	90.00	29.0000	25	83.00	24.0000	60	11.0%	0.27 [-0.20; 0.74]
Temimović 2019	113.15	44.9000	36	101.31	33.0000	44	11.1%	0.30 [-0.14; 0.75]
Total (95% CI)			195			220	71.3%	0.65 [0.08; 1.22]
Heterogeneity: $Tau^2 = 0.2873$; $Chi^2 = 25.99$, df = 6 ($P < 0.01$); $I^2 = 77\%$								
CMR								
Perseghin 2005	77.90	15.6000	38	83.00	11.0000	16	10.2%	-0.35 [-0.94; 0.24]
Barbosa 2021	85.00	16.0000	44	67.00	11.0000	10	9.4%	1.16 [0.44; 1.89]
Qi 2022	58.80	10.5000	16	42.80	7.9000	21	9.1%	1.72 [0.95; 2.49]
Total (95% CI)			98			47	28.7%	0.82 [-1.85; 3.50]
Heterogeneity: $Tau^2 = 1.0445$; $Chi^2 = 20.4$, df = 2 ($P < 0.01$); $I^2 = 90\%$								
**Total (95% CI)**			**293**			**267**	**100.0%**	**0.70 [0.19; 1.21]**
**Prediction interval**								**[-0.86; 2.25]**

Heterogeneity: $Tau^2 = 0.4061$; $Chi^2 = 46.48$, df = 9 ($P < 0.01$); $I^2 = 81\%$
Test for subgroup differences: $Chi^2 = 0.07$, df = 1 ($P = 0.79$)

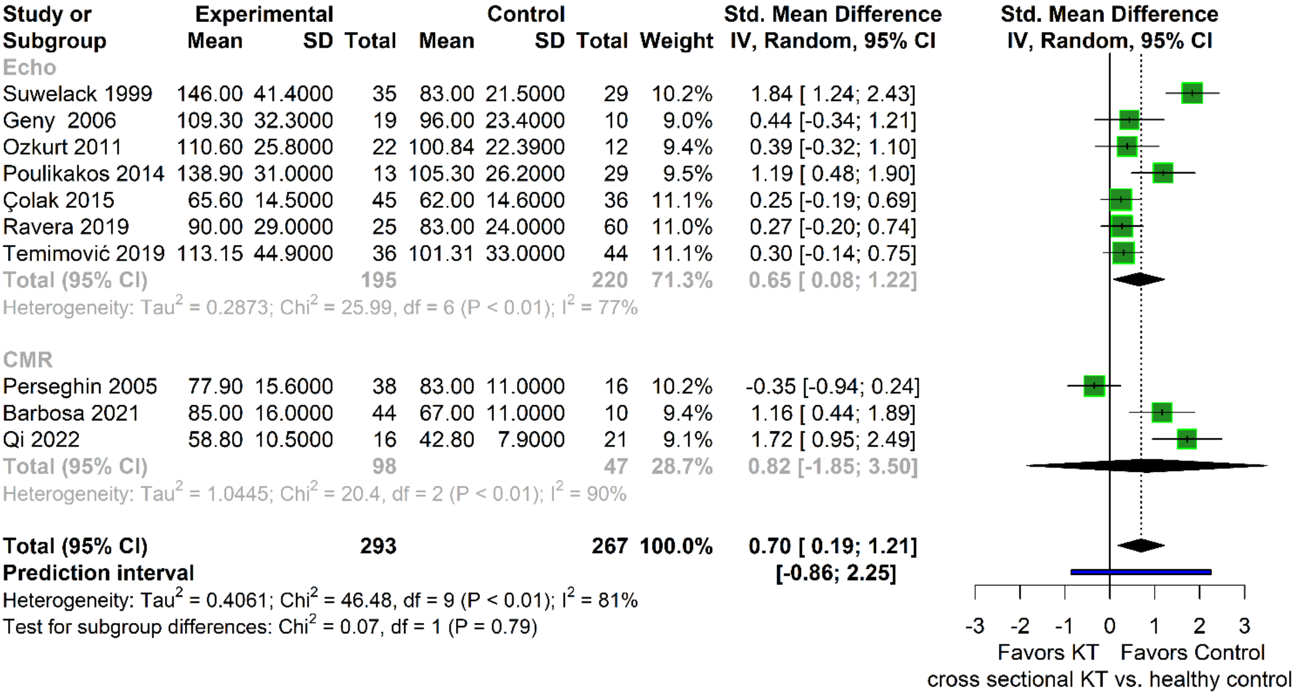

Figure S12. Forest plot illustrating cross-sectional studies with renal recipients and healthy participants

Estimated effect sizes for LVMI changes are presented as standardized mean difference and 95% confidence interval. Heterogeneity analysis using I^2 and Tau^2 is illustrated. A significant higher left ventricular mass index is observed in renal recipients compared to healthy individuals (0.70 g/m2 [0.19; 1.21]; P < 0.01), regardless of imaging procedures.
KT: kidney transplantation
